# Profiling Oncogenic Germline Mutations in Unselected Chinese Lung Cancer Patients

**DOI:** 10.3389/fonc.2021.647598

**Published:** 2021-04-07

**Authors:** Jie Yang, Hefei Li, Ben Li, Wei Li, Qiang Guo, Ling Hu, Zizheng Song, Bin Zhou

**Affiliations:** ^1^ Radiotherapy Department, The Fourth Hospital of Hebei Medical University, Shijiazhuang, China; ^2^ Department of Thoracic Surgery, Affiliated Hospital of Hebei University, Baoding, China; ^3^ Department of Medical Oncology, Affiliated Hospital of Hebei University, Baoding, China

**Keywords:** germline mutation, oncogenic gene, lung cancers, likely pathogenic/pathogenic variant, variants of uncertain significance

## Abstract

**Introduction:**

Emerging evidence has suggested that inherited factors are also involved in lung cancer development. However, most studies focused on well-elucidated cancer predisposition genes, the majority of which are tumor suppressor genes. The profile of germline mutations in oncogenic driver genes remains unrevealed, which might also provide potential clinical implications for lung cancer management.

**Methods:**

Sequencing data from 36,813 unselected lung cancer patients who underwent somatic mutation profiling were retrospectively reviewed. All recruited patients had matched white blood cell samples sequenced in parallel using a capture-based panel including eight key lung cancer driver genes (*epidermal growth factor receptor* (*EGFR), anaplastic lymphoma kinase (ALK), MET proto-oncogene, receptor tyrosine kinase (MET), Kirsten rat sarcoma viral oncogene homolog (KRAS)*, *Erb-B2 receptor tyrosine kinase 2(ERBB2), ROS proto-oncogene 1, receptor tyrosine kinase (ROS1), ret proto-oncogene (RET)*, and *B-Raf proto-oncogene, serine/threonine kinase* (*BRAF)*). Likely pathogenic/pathogenic (LP/P) variants were called according to the classification criteria of the American College of Medical Genetics and Genomics. Variants of uncertain significance (VUS) located in the kinase domains of driver genes and occurring recurrently (n ≥3) were also included for further analyses.

**Results:**

Seven different LP/P variants in *EGFR*, *MET*, or *RET* were identified in 0.03% of lung cancer patients (n = 14) and 25 different VUS in the kinase domains of seven driver genes (except *KRAS*) were found with a prevalence of 0.3% (n = 117).Collectively, germline mutations were most frequently seen in *ROS1* (n = 31, 0.084%), followed by *MET* (n = 23, 0.062%), *EGFR* (n = 22, 0.06%), *ALK* (n = 22, 0.06%) and *RET* (n = 17, 0.046%). LP/P variants and VUS fell the most commonly in *EGFR* (n = 10, 72%) and *ROS1* (n = 31, 26%), respectively. Of the 10 patients with *EGFR* LP/P germline mutation, 70% also acquired somatic *EGFR* driver mutation exon21 p.L858R or exon19 deletion at baseline; while the three patients with pathogenic germline *RET* mutation displayed distinct baseline somatic profiles of rare *EGFR* mutation or *KRAS* exon2 p.G12C. We discovered 11 germline mutations that also occurred somatically, including four LP/P variants and seven VUS.

**Conclusion:**

We present the first study to systemically characterize the germline mutation in oncogenic driver genes in a large cohort of unselected patients with lung cancers.

## Introduction

Lung cancer is the most prevalent cancer worldwide and the leading cause of cancer-related mortality (1), which is partially attributable to its diagnosis at advanced stages. Environmental factors, such as tobacco exposure and air pollution, are generally considered as major etiological factors for lung tumorigenesis (2). However, mounting evidence has suggested that inherited factors are also involved in lung cancer development. Rare familial patterns of lung cancers have been reported in sporadic case reports, in concordance with the autosomal dominant inheritance (3–7). Germline p.T790M and p.V843I in *epidermal growth factor receptor (EGFR)* and *parkin RBR E3 ubiquitin protein ligase (PARK2)* loss-of-function mutations have been identified in these families and suggested to confer the high susceptibility to lung cancer. More recently, with the introduction of next-generation sequencing (NGS) in clinical settings, studies with larger cohorts have also been performed to systemically investigate the prevalence of pathogenic germline mutations in sporadic lung cancers. The vast majority of these studies focused on previously identified cancer predisposition genes (mostly tumor suppressor genes) and demonstrated that lung cancer patients, especially those with adenocarcinoma, harbor enriched germline mutations in DNA repair genes (8–11).

Oncogenic driver mutations constitutively activating signaling pathways can result in uncontrolled cell growth and proliferation, which is an essential mechanism underlying carcinogenesis. Previous studies of lung cancer primarily aimed to identify driver genes somatically and have revealed a number of oncogenic driver mutations especially in non-small cell lung cancer (NSCLC), consisting of alterations in *EGFR*, *Kirsten rat sarcoma viral oncogene homolog (KRAS)*, and *anaplastic lymphoma kinase (ALK)* etc. (12, 13). These genetic alterations offer specific molecular therapeutic targets. Several such targeted therapies, EGFR and ALK inhibitors for instance, have demonstrated promising clinical efficacy in NSCLC patients harboring the corresponding mutation. Thus, molecular testing for these driver genes has become the standard of care for the management of advanced NSCLC (14). Unlike the well-characterized profiles of somatic driver mutations in lung cancer, limited efforts have been invested to elucidate germline mutations in these driver genes, which however might also provide potential clinical implications for lung cancer management, such as risk assessment, prevention and targeted therapy (15, 16).

In the present study, we retrospectively reviewed the genomic data of 36, 813 unselected Chinese patients with lung cancers, aiming to investigate the prevalence and spectrum of germline mutations in the key lung cancer driver genes in this population.

## Materials and Methods

### Study Design and Patients’ Information

**Figure 1:**
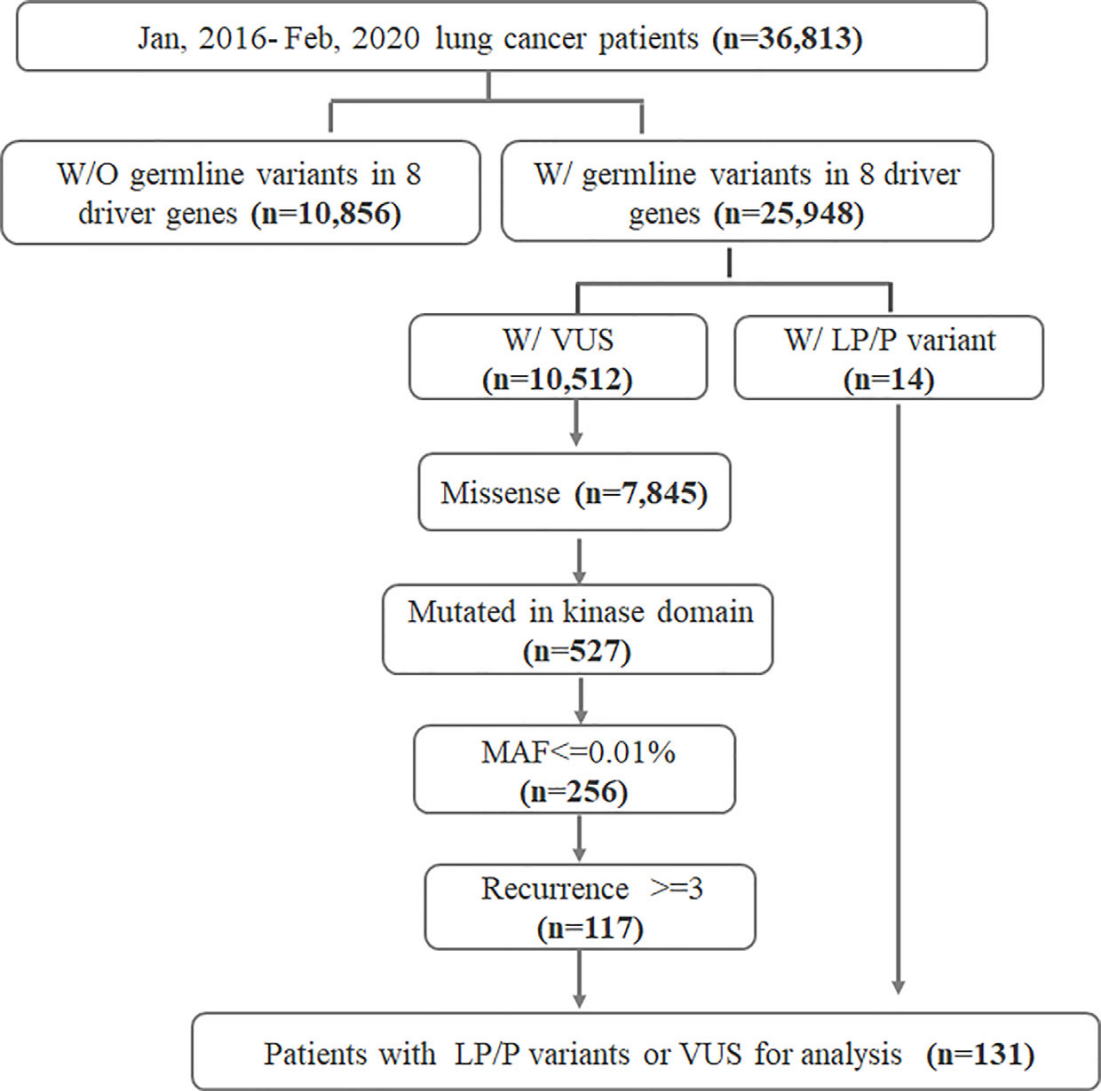
Flowchart of the study design. W/, with; W/O, without; LP, likely pathogenic; P, pathogenic; VUS, variants of uncertain significance; MAF, minor allele frequency; eight driver genes include *EGFR*, *ALK*, *MET*, *KRAS*, *ERBB2*, *ROS1*, *RET*, and *BRAF*.

We retrospectively reviewed the sequencing data from 36,813 lung cancer patients who underwent somatic mutation profiling for treatment selection and genetic testing from January 2016 to February 2020. Recruited patients provided matched white blood cell (WBC) samples for sequencing in parallel for the purpose of germline mutation filtration. Samples were sequenced with a capture-based panel including the 8 key lung cancer driver genes (*EGFR*, *ALK*, *MET proto-oncogene*, *receptor tyrosine kinase (MET)*, *KRAS*, *Erb-B2 receptor tyrosine kinase 2 (ERBB2)*, *ROS proto-oncogene 1*, *receptor tyrosine kinase (ROS1)*, *ret proto-oncogene (RET)*, and *B-Raf proto-oncogene*, *serine/threonine kinase* (*BRAF)*) (Burning Rock, Guangzhou, China). Of the 36,813 patients, 10,856 had no germline variants detected in any of the eight driver genes and were excluded. Of the remaining 25,948 patients harboring driver gene germline variant(s), 14 patients were identified with a likely pathogenic/pathogenic (LP/P) germline variant. Germline variants of uncertain significance (VUS) were identified in 10,512 patients. We further screened 527 patients whose VUS were missense variants occurring in the kinase domains from among the 10,512 patients. Other inclusion criteria for VUS consisted of major allele frequency (MAF) ≤0.01% and recurrence count ≥3 in the cohort. Ultimately, a total of 131 patients were included for further analysis (117 with VUS and 14 with LP/P variants). The overall study design was illustrated in **Figure 1**. Results obtained in this study were considered research and were not returned to study participants or their clinicians for decision making. Patients were unselected for age or personal and family history of cancers. Patients’ sex, age at diagnosis, clinical diagnosis, and stage were obtained from medical records. The study was conducted in accordance with the Declaration of Helsinki (as revised in 2013). The study was approved by the institutional review board (IRB) of The Fourth Hospital of Hebei Medical University. Informed consent was not required due to the retrospective nature of the study.

### Sequencing and Germline Variant Calling

DNA was sequenced on Nextseq500 sequencer (Illumina, Inc., USA) with paired-end reads in a Clinical Laboratory Improvement Amendments (CLIA)/CAP-certified laboratory using a capture-based panel at least including the eight key lung cancer driver genes (*EGFR*, *ALK*, *MET*, *KRAS*, *ERBB2*, *ROS1*, *RET*, and *BRAF*) (Burning Rock Biotech, Guangzhou, China). The sequencing depth was 1,000× for both tissues and their matched WBC samples, as well as 10,000× and 5,000× for plasmas and their matched WBC samples, respectively. Trimmomatic (v0.36) was used to clean sequencing reads. Burrows-Wheeler Aligner (17) was used to map all cleaned reads to GRCh37/hg19 genome with -Y -M parameters. Copy number variation and indel variant calling was performed using vardict 1.5.1. Variants with depth <50× or mutated allele reads <8× were filtered out as low quality. Variants with allele frequency >10% in both WBC and tumor were determined as germline.

Variants with population frequencies over 0.1% in the ExAC, 1,000 Genomes, dbSNP, or ESP6500SI-V2 databases were grouped as single nucleotide polymorphisms and excluded from further analysis. The reported mutations were further confirmed with ClinVar databases. Variant annotations were aggregated by Intervar (18) from multiple databases, prediction tools, and publications at a single site. In the absence of clinical data and *in vitro* functional assay, *in silico* prediction was performed using algorithms that assess phylogenetic conservation and the likelihood of severe physiochemical alterations in the protein structure or function. All genetic annotations and nomenclature were based on GRCh37/hg19 build. The variants were classified according to the American College of Medical Genetics and Genomics (ACMG) recommendations for standards of interpretation and reporting of sequence variations as follows: pathogenic (Class 5), likely pathogenic (Class 4), variants of uncertain significance (Class 3), likely benign (Class 2), and benign (Class 1) (19).

### Statistical Analysis

All data were analyzed using R software. Patient characteristics and sequencing results were summarized with descriptive statistics, including medians, means, and standard deviations for continuous data. Differences in groups were compared using Fisher’s exact test, paired two-tailed Student’s t-test or analysis of variance, as applicable. P <0.05 was considered statistically significant.

## Results

### Characteristics of Patients

We retrospectively reviewed the genomic sequencing data of 36,813 lung cancer patients, profiled from January 2016 to February 2020. The cohort had a median age of 62 years at diagnosis, with 49.7% male, 42.2% female, and 8.1% without sex information (**Table 1**). Of the 36,813 patients, 24,977 (67.8%) were diagnosed with adenocarcinomas, 2,131 (5.8%) with squamous carcinomas and 9,262 (25.2%) with small cell carcinomas. A total of 8,200 patients (22.3%) had early diseases (stages I–IIIA) while 23,778 (64.6%) were at late stages (stages IIIB–IV).

**Table 1:** Clinicopathological characteristics of the cohort.

Characteristic	All(n = 36,813)	Germline mutation carriers(n = 131)	*P*
**Age, years**			
median [IQR]	62.00 [54.00, 69.00]	62.00 [54.75, 68.25]	0.76
**Sex, n (%)**			0.346
Male	18295 (49.7)	73 (55.7)	
Female	15525 (42.2)	50 (38.2)	
Unknown	2993 (8.1)	8 (6.1)	
**Histology, n (%)**			0.923
Lung adenocarcinoma	24977 (67.8)	92 (70.2)	
Lung squamous carcinoma	2131 (5.8)	7 (5.3)	
Small cell lung carcinoma	9262 (25.2)	31 (23.7)	
Others	443 (1.2)	1 (0.8)	
**Clinical Stage, n (%)**			0.029
I-IIIA	8200 (22.3)	33 (25.2)	
IIIB-IV	23778 (64.6)	91 (69.5)	
Unknown	4835 (13.1)	7 (5.3)	

A total of 14 out of 36,813 lung cancer patients (0.03%) were identified with an LP/P germline variant, while 117 patients (0.3%) were detected with germline VUS that met the inclusion criteria (**Figure 1**). The clinical characteristics of the 131 germline mutation carriers were also summarized in **Table 1**, which demonstrated no significant difference compared with the whole cohort except for the clinical stage.

### The Prevalence and Spectrum of Germline Mutations in Driver Genes

A total of 32 different germline mutations (seven LP/P or 25 VUS) were identified in driver genes from 131 lung cancer patients. The majority of patients (127/131) harbored a heterogeneous mutation and only one patient (P73) harbored a homozygous *ROS* VUS (**Table S1**). Of note, patient P97 carried a heterogeneous VUS in *MET* and *ERBB2*, respectively, while patient P126 had a heterogeneous VUS in *ALK* and a homozygous VUS in *RET* (**Table S1**).

**Figure 2:**
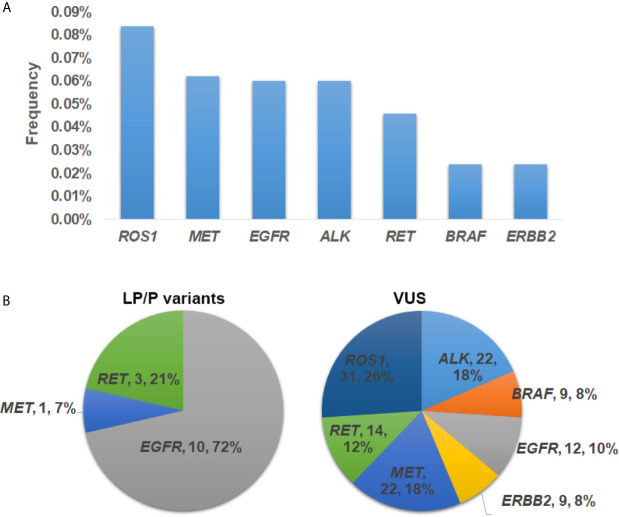
The prevalence and distribution of germline mutations in driver genes. **(A)** The prevalence of putative pathogenic mutations in each driver gene. **(B)** Distribution of likely pathogenic/pathogenic (LP/P) variants and variants of uncertain significance (VUS) in each driver gene.

Collectively, germline mutations occurred the most commonly in *ROS1* (n = 31, 0.084%), followed by *MET* (n = 23, 0.062%), *EGFR* (n = 22, 0.06%), *ALK* (n = 22, 0.06%) and *RET* (n = 17, 0.046%) (**Figure 2A**). Both *ERBB2* and *BRAF* had a mutation frequency of 0.024% (n = 9). Specifically, 72% of the LP/P variants occurred in *EGFR* gene (n = 10) (**Figure 2B**). The remaining LP/P variants were found in *RET* (n = 3, 21%) and *MET* (n = 1, 7%). On the other hand, VUS fell the most commonly in *ROS1* (n = 31, 26%), followed by *MET* (n = 22, 18%) and *ALK* (n = 22, 18%) (**Figure 2C**). Besides, SUV were also identified in *RET* (n = 14, 12%), *EGFR* (n = 12, 10%), *ERBB2* (n = 9, 8%) and *BRAF* (n = 9, 8%). Of note, we did not identify any putative pathogenic germline variants from *KRAS*.

**Figure 3:**
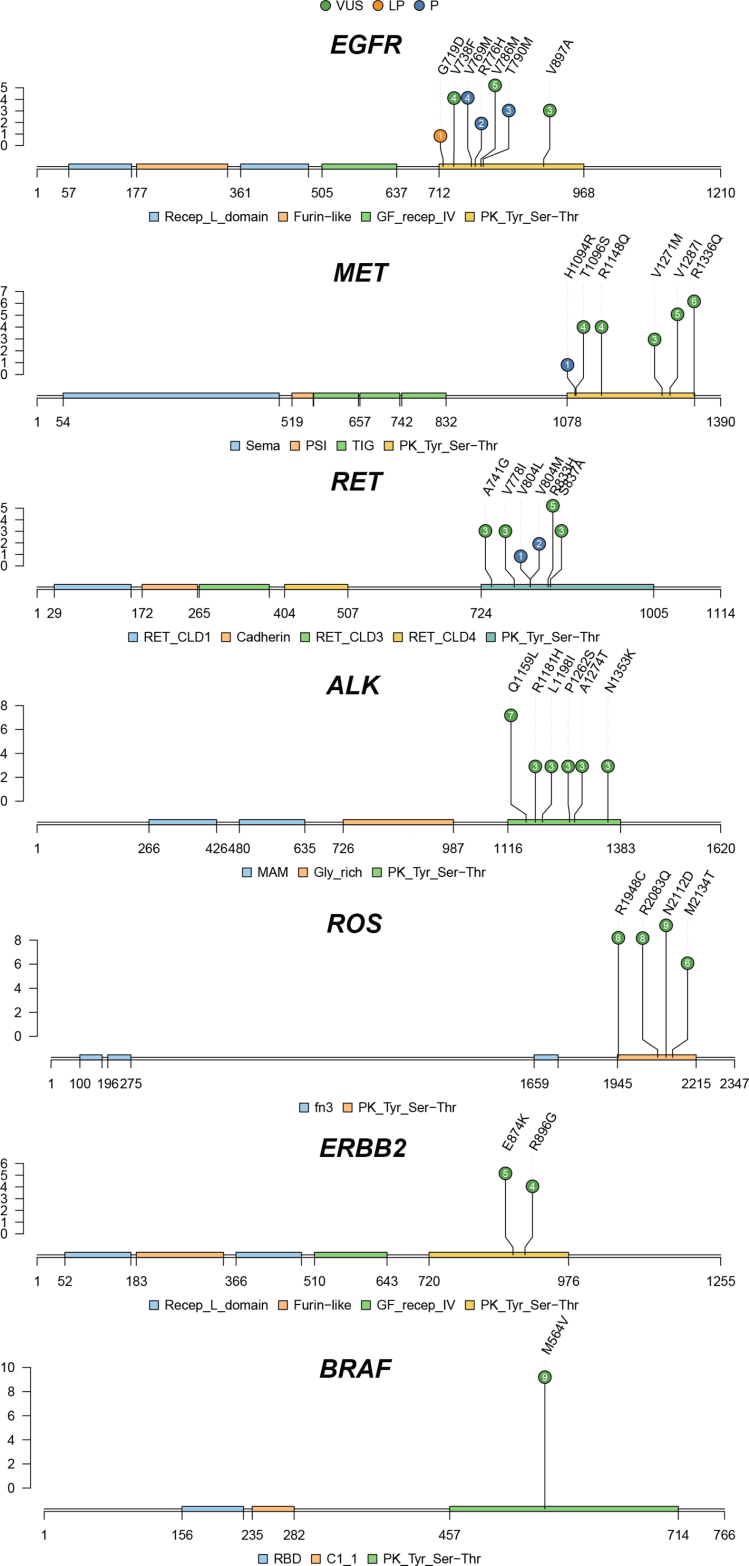
The spectrum of germline mutations in driver genes.

A total of seven different germline mutations were identified in *EGFR* (**Figure 3**), including three pathogenic variants (exon20 p.V769M, n = 4; exon20 p.T790M, n = 3; exon20 p.R776H, n = 2), one likely pathogenic variant (exon20 p.G719D, n = 1) and three VUS (exon20 p.V786M, n = 5; exon19 p.V738F, n = 4; exon22 p.V897A, n = 3). *MET* exon16 p.H1094R, the only pathogenic variant found in *MET*, was detected in one patient (**Figure 3**). Besides, five VUS were also identified in the *MET* gene, with exon21 p.R1336Q (n = 6) and exon20 p.V1287I (n = 5) being the most common ones. For the *RET* gene, we discovered two pathogenic variants (exon14 p.V804M, n = 2; exon14 p.V804L, n = 1), which are the driver mutations commonly seen in thyroid cancer. Four VUS were identified in *RET*, with exon14 p.R833H occurring the most frequently (n = 5). We identified six, four, two, and one VUS in *ALK*, *ROS*, *ERBB2* and *BRAF*, respectively. Among them, *BRAF* exon13 p.M564V (n = 9), *ROS* exon40 p.N2112D (n = 9), exon36 p.R1948C (n = 8), exon40 p.R2083Q (n = 8), and *ALK* exon22 p.Q1159L (n = 7) were hotspot variants.

### The Baseline Somatic Genomic Profile in LP/P Germline Mutation Carriers

We also investigated the baseline somatic genomic profiles of the 14 patients harboring LP/P germline mutations. Of the 10 patients with *EGFR* LP/P germline mutations, 70% (n = 7) also acquired somatic *EGFR* driver mutation exon21 p.L858R or exon19 deletion at baseline, while one patient acquired *EGFR* exon20 p.G719A and two patients lacked driver mutations at baseline (**Table 2**). The patient with pathogenic germline *MET* exon16 p.H1094R also acquired *EGFR* exon21 p.L858R at baseline. However, the three patients harboring pathogenic *RET* mutations did not acquire common *EGFR* driver mutation at baseline. Instead, one patient had two rare *EGFR* mutations exon21 p.L858M and exon20 p.V774M, one had *KRAS* exon2 p.G12C, and one was driver mutation-negative.

**Table 2:** Clinical and genomic information for 14 patients harboring LP/P germline mutations in eight key driver genes.

Patient	Sex	Age	Histology	Clinical Stage	Germline mutation	Het/Homo	Classification	Baseline driver gene somatic alteration
P1	F	49	Adeno	IVa	*EGFR* exon20 p.V769M	Het	P	*EGFR* exon21 p.L858R, *EGFR* amp; *MET* amp
P2	F	55	Adeno	IVb	*EGFR* exon20 p.V769M	Het	P	*EGFR* exon19 del;
P3	F	60	Adeno	IVb	*EGFR* exon20 p.V769M	Het	P	*EGFR* exon20 p.G719A, *EGFR* amp
P4	M	57	Adeno	IVa	*EGFR* exon20 p.V769M	Het	P	–
P5	F	57	Adeno	IIIa	*EGFR* exon20 p.T790M	Het	P	*EGFR* exon21 p.L858R
P6	F	52	Adeno	IVa	*EGFR* exon20 p.T790M	Het	P	*EGFR* exon21 p.L858R
P7	F	65	Adeno	IVb	*EGFR* exon20 p.T790M	Het	P	*EGFR* exon19 del, *EGFR* amp; *ERBB2* intron variant; *KRAS* del
P8	M	61	Adeno	IVb	*EGFR* exon20 p.R776H	Het	P	*EGFR* exon21 p.L858R
P9	M	48	Adeno	IIIa	*EGFR* exon20 p.R776H	Het	P	–
P10	M	55	Adeno	IVa	*EGFR* exon20 p.G719D	Het	LP	*EGFR* exon21 p.L861R
P11	F	53	Adeno	IVa	*RET* exon14 p.V804M	Het	P	*EGFR* exon20 p.V774M, exon21 p.L858M;
P12	M	73	Adeno	IVb	*RET* exon14 p.V804M	Het	P	*KRAS* exon2 p.G12C
P13	M	58	Adeno	IVa	*RET* exon14 p.V804L	Het	P	–
P14	F	78	Adeno	II	*MET* exon16 p.H1094R	Het	P	*EGFR* exon21 p.L861R

F, female; M, male; Het, heterozygous; Homo, homozygous; P, pathogenic; LP, likely pathogenic; amp, amplification; del, deletion.

### Germline Mutations Also Occurred Somatically

We discovered 11 germline mutations that also occurred somatically. Somatic *EGFR* gatekeeper mutation exon20 p.T790M was seen in 3164 patients (8.65%) (**Table 3**). Somatic *EGFR* oncogenic mutations exon20 p.R776H and exon20 p.G719D were observed in 31 and five patients, respectively. *RET* exon14 p.V804L, a common thyroid cancer driver mutation, was also detected as somatic in two lung cancer patients. Besides the four LP/P variants, seven VUS were also found somatically mutated in patients, including *MET* exon17 p.R1148Q and exon21 p.R1336Q, *ROS1* exon36 p. R1948C, *BRAF* exon13 p.M564V, *ALK* exon23 p.L1198I, *ERBB2* exon21 p.E874K, and *EGFR* exon22 p.V897A, suggesting their pathogenicity in tumorigenesis.

**Table 3:** List of germline P/LP mutations or VUS that also occurred somatically.

Gene	Exon	Variant	No. of germline	No. of somatic	ACMG classification
*EGFR*	20	p.T790M	3	3,164	P
*EGFR*	20	p.R776H	2	31	P
*EGFR*	20	p.G719D	1	5	LP
*MET*	17	p.R1148Q	4	4	VUS
*ROS1*	36	p.R1948C	8	2	VUS
*MET*	21	p.R1336Q	6	2	VUS
*RET*	14	p.V804L	1	2	P
*BRAF*	13	p.M564V	9	1	VUS
*ALK*	23	p.L1198I	3	1	VUS
*ERBB2*	21	p.E874K	5	1	VUS
*EGFR*	22	p.V897A	3	1	VUS

P, pathogenic; LP, likely pathogenic; VUS,variants of uncertain significance.

### The Association of Mutation Status With Clinical Characteristics

**Figure 4:**
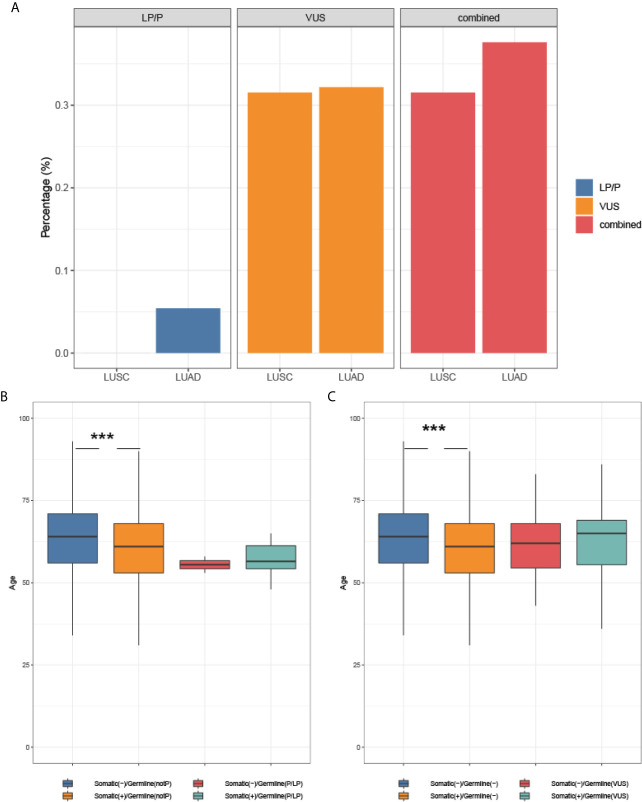
The association of mutation status with histology and onset age. **(A)** Germline mutation and histology; **(B)** The association of somatic and LP/P mutations with onset age; **(C)** The association of somatic mutation and VUS with onset age. *** indicates a P-value <0.001.

Next, we investigated the association of germline and somatic driver mutation status with histology and onset age. As shown in **Figure 4A**, the prevalence of LP/P variants and VUS in adenocarcinomas was not significantly different from that in squamous carcinoma, though LP/P germline variants were only identified from adenocarcinomas. In the subset of patients without LP/P germline mutation, somatic driver mutation was significantly associated with an earlier age at diagnosis (61.0 years vs. 64.0 years, p <0.001, **Figure 4B**). Patients with LP/P germline mutations showed a median age of 56.5 and 55.5 years in the subsets with and without concomitant somatic driver mutations, respectively, compared with the median age of 64.0 years in germline and somatic mutation-negative patients (p = 0.11; p = 0.19). Similarly, patients harboring VUS neither display significantly earlier onset age compared with VUS non-carriers regardless of somatic status (**Figure 4C**), most likely attributable to the small number of germline mutation carriers identified in the study.

## Discussion

To the best of our knowledge, this is the first study to systemically explore oncogenic germline mutations in sporadic lung cancers. We identified seven different LP/P variants in three driver genes (*EGFR, MET*, and *RET*) in 0.03% of unselected lung cancer patients. We also identified 25 different VUS in the kinase domains of driver genes (except *KRAS*) that were recurrently detected at least in three patients, with a prevalence of 0.3%. A previous study in 12,833 Chinese lung cancer patients focusing on *EGFR* and *ERBB2* has revealed a prevalence of 0.11 and 0.01% for germline mutations in the former and the latter, respectively (15). In our cohort, *EGFR* and *ERBB2* displayed a similar germline mutation rate of 0.06% (P = 0.07) and 0.024% (P = 0.35), respectively. However, the spectrum of mutations differs between two studies: of the eight *EGFR* germline mutations identified from Lu et al., only p.T790M and p.V786M were detected in our cohort. The germline p.T790M initially has been reported in familial cohorts (3, 20). A recent study performed by Dana–Farber Institute revealed a prevalence of 0.15% for germline p.T790M in 31,414 patients with *EGFR*-mutant (including T790M) NSCLCs (21). In comparison, germline p.T790M was observed in ~0.008% of unselected Chinese lung patients [Figure 3 and Lu et al. (15)]. These observations indicate enrichment of germline p.T790M in *EGFR*-positive tumors. However, the ethnic difference might also contribute in part to the discrepant p.T790M frequencies, since it has been suggested that germline mutations in lung cancers are more common among patients from Caucasian than from Eastern Asian (22). Besides p.T790M, the pathogenic germline *EGFR* exon20 p.R776H and p.V769M identified in our cohort have also been reported in sporadic lung cancer cases (23, 24); whereas pathogenic germline mutations *MET* exon16 p.H1094R, *RET* exon14 p.V804M and p.V804L, and likely pathogenic *EGFR* exon20 p.G719D are reported in lung cancer for the first time. *MET* p.H1094R has previously been described in papillary renal cell carcinoma (25). Germline mutations at codon 804 in *RET* are commonly identified in patients with multiple endocrine neoplasia type 2 and confer an elevated lifetime risk of medullary thyroid carcinoma (26, 27).

We also observed that approximately 70% of the oncogenic LP/P germline mutation carriers (**Table 2**) acquired somatic *EGFR* mutations at the time of diagnosis, comparable with that of 66.7% reported in patients with *EGFR/ERBB2* germline mutations (15). The observation suggests that the tumorigenesis in patients with germline oncogenic mutation is more likely to be driven by *EGFR* mutation. Of note, the vast majority of *EGFR* or *MET* germline mutation carriers in our study harbored the common *EGFR* driver mutation exon19 deletion or exon21 p.L858R, while *RET* germline mutation carriers displayed distinct baseline somatic profiles of rare *EGFR* mutation or *KRAS* exon2 p.G12C, which might indicate distinctive mechanisms underlying tumorigenesis in *RET*–mutant patients.

It has been suggested that patients harboring *EGFR* germline mutation but without any known somatic driver mutations might also benefit from EGFR TKIs. Lu et al. described a patient with a *EGFR* germline p.L844V who responded to afatinib, achieving a PFS of 13 months (15). Tibaldi et al. reported an NSCLC patient harboring a germline p.T790M who achieved partial response (PR) to gefitinib with a PFS of 45 months, which also suggests that the inherited p.T790M mutation is not necessarily predictive of resistance to first-generation EGFR TKI (28). The predictive and prognostic values of the oncogenic germline mutations identified in our study merit systemic investigation in the future and the results might facilitate the stratification of lung cancer patients for targeted therapy.

Germline mutation in cancer predisposition genes often confers an earlier onset in several cancers including breast and colorectal cancers (29, 30). However, the role of germline mutations in lung cancer lacks thorough investigation thus remains elusive. Hu et al. reported a positive association between germline *BRCA1/2* mutation and early onset in NCSLC (11). In the present study, although the numeric value of median onset age of germline oncogenic mutation-carriers was younger than that of non-carriers (55.5 years vs. 64 years), we did not observe a significant association (p = 0.19), most likely due to the small number of germline oncogenic mutation carriers identified in our study. However, we found a positive association between somatic driver mutation and earlier onset (61.0 years vs. 64.0 years, p <0.001) in non-germline carriers, but such phenomenon was not present in the context of germline oncogenic mutation, which might in part suggest the role of these germline oncogenic mutations in tumorigenesis.

In conclusion, we present the first study to systemically characterize the germline mutation in oncogenic driver genes in a large cohort of unselected patients with lung cancer. Our findings may provide potential clinical implications for lung cancer management.

## Data Availability Statement

The original contributions presented in the study are included in the article/**Supplementary Material**. Further inquiries can be directed to the corresponding authors.

## Ethics Statement

The studies involving human participants were reviewed and approved by The Fourth Hospital of Hebei Medical University. Written informed consent for participation was not required for this study in accordance with the national legislation and the institutional requirements.

## Author Contributions

JY, HL, ZS, and BZ contributed to the conception or design of the work. BL, WL, and QG contributed to the acquisition of data. JY, HL, and LH contributed to the analysis of data. ZS and BZ contributed to the interpretation of the data. JY and HL drafted the MS. All other authors revised the MS. All authors contributed to the article and approved the submitted version.

## Conflict of Interest

The authors declare that the research was conducted in the absence of any commercial or financial relationships that could be construed as a potential conflict of interest.
